# Compliance of large language model generated meal plans with polish dietary recommendations: a real-world evaluation under average user conditions—3(LM)Diet study

**DOI:** 10.3389/fpubh.2026.1949700

**Published:** 2026-09-14

**Authors:** Hubert Dobrowolski

**Affiliations:** School of Medical & Health Sciences, VIZJA University, Warsaw, Poland

**Keywords:** artificial intelligence, diets analysis, healthy eating, large language models, nutritional recommendations

## Abstract

**Introduction:**

Diet is one of the key factors influencing health. Adherence to the principles of a healthy diet determines whether many diseases occur or are prevented. Numerous guidelines and recommendations have been issued to facilitate the adoption of healthy eating practices. However, putting them into practice can be problematic. In recent years, increasing attention has been paid to large language models (LLMs). A growing number of reports also point to the risks arising from the uncritical adoption by the general public of health recommendations generated by LLMs.

**Aim:**

The aim of this study was to evaluate whether meal plans generated by LLMs comply with Polish healthy eating recommendations under real-world average-user conditions.

**Materials and methods:**

The three widely used LLMs – ChatGPT, Gemini and Copilot – were used to generate diets. Each model generated 35 days of meal plans across three specified energy levels: 2,000 kcal, 2,500 kcal and 3,000 kcal (in total, each model generated 105 days of meal plans). The menus were assessed for compliance with the recommendations based on the average number of guidelines met each day and the total number of guidelines met.

**Results:**

Copilot achieved the highest overall compliance score (median 7/9 recommendations per day), significantly outperforming ChatGPT (median 6/9; *p* = 0.002) and Gemini (median 5/9; *p* < 0.001). Compliance was generally independent of the target energy level. All models consistently limited red and processed meat intake and usually met recommendations regarding fruit and vegetable consumption, sweets, fats, and dairy products. However, adherence was poor for wholegrain products. None of the evaluated models achieved full compliance with all tested national dietary recommendations.

**Conclusion:**

Diets generated by LLMs demonstrated moderate compliance with Polish dietary recommendations, although important shortcomings remained. While AI-generated meal plans may encourage healthier dietary patterns in some areas, they cannot yet be considered fully consistent with national dietary guidance. LLM-generated meal plans should be interpreted with caution and should not replace professional dietary counseling.

## Introduction

Diet is one of the most important factors affecting health. A properly balanced diet is an effective means of prevention and treatment, while an unbalanced food intake is a risk factor linked to many diseases (1–3). However, for a diet to be beneficial to health, it must meet certain criteria, which can be confusing for those without the necessary knowledge. Studies indicate that adequate nutritional knowledge and awareness are linked to the quality of one’s diet (4–6). To help people follow the principles of a healthy diet, many countries have issued dietary guidelines for their citizens (7, 8). Recommendations have also been issued by scientific societies, as well as guidelines on healthy diet issued by the World Health Organisation (9). National guidelines, however, are better tailored to the needs of a given society, as they consider specific factors that general guidelines are unable to address, given the diversity of the population (10–12). Adherence to regional guidelines may therefore be a more appropriate way of improving one’s diet than following general, international guidelines.

Among the numerous national recommendations, Poland has also issued its own recommendations (13). Polish healthy eating guidelines are presented in the form of a healthy eating plate, which visually illustrates a plate with the appropriate proportions of different food groups, as well as general recommendations such as limiting red meat, increasing the proportion of wholegrains, and ensuring adequate hydration (14). To support the implementation of the guidelines, the National Institute of Public Health – National Institute of Hygiene, which developed these recommendations, has also issued healthy eating guidelines that enable the principles to be gradually incorporated into daily life in three steps. These recommendations are structured from the least (step 1) to the most (step 3) demanding, to facilitate gradual implementation.

Although these recommendations do not require specialist skills to be put into practice, as their content is accessible to the average person’s needs and knowledge, a lack of practice, conviction or the desire to vary dietary plans may cause difficulties in implementing them. Numerous studies have highlighted barriers to the implementation of such recommendations among the general public (15–18). It is only natural that people facing these difficulties might seek advice on how to implement the recommendations from external sources, such as specialists or even the internet.

One possible solution in this area are Large Language Models (LLMs) which are becoming increasingly popular (19). These are tools designed to understand and generate text that resembles human writing, based on specified prompts. The more effective and refined the prompt, the better, more precise and more accurate the response (20, 21). However, imprecisely worded questions and an uncritical approach to the answers received can lead to adverse consequences, particularly in the health sector (22–24). However, the average user may lack the necessary knowledge and experience to formulate a query correctly and then properly verify it.

The 3(LM)Diet project aims to evaluate diets generated by LLMs. This evaluation is multi-dimensional, covering both quantitative and qualitative assessments of meal plans, their compliance with standards and recommendations, as well as relevant issues – such as the expense of the diet, the sustainability of meal plans and environmental considerations. However, the aim of the project is not to assess the full potential of language models, but rather the practical implications of their use – by the average user without specialist knowledge or the ability to verify information. This project conducts a thorough analysis of the real benefits and risks arising from the use of these tools by the average user. An important feature is that the analyses are grounded in the realities of a specific country.

Given the importance of dietary guidelines in shaping a healthy diet, the difficulties an average user may encounter in applying these guidelines, and the growing popularity of LLMs in modern society, the aim of this study was to assess the alignment of meal plans generated by the most popular language models with current national dietary recommendations, in the real-life context of these diets being created by an average user without specialist knowledge or skills.

## Materials and methods

### Large language models used

Three models were used to generate the menus: ChatGPT (GPT-5.5, accessed 11.05.26) (25), Gemini (Gemini 2.5 Flash, accessed 12.05.26) (26), and Microsoft 365 Copilot (based on the GPT-5 model, accessed 15.05.26) (27). All prompts were executed between 11 May 2026 and 25 May 2026. No intentional model updates were introduced by the investigators during data collection. The selection of these models was based on their popularity among modern users, their ease and widespread availability, and the recognition of the brands behind these tools. Prompts were issued to the models on accounts that had not previously generated information related to diet or a healthy lifestyle to minimize the conversation history or personalization effects. The accounts were therefore not burdened with information or queries regarding diet, a healthy lifestyle or other health-related issues. Free accounts made it possible to simulate users with an non-expert approach, and therefore more likely to be unfamiliar with the correct method of creating prompts. All requests and generations took place in Warsaw, Poland.

### Prompts used for generating diets

All commands were issued in Polish. In the initial task, the language models were asked to generate 35-day meal plans. The prompt specified in detail all the necessary guidelines and characteristics that the generated meal plan should meet, including energy content and compliance with nutritional standards and recommendations. The generated meal plans had to be balanced on a weekly basis to take into account the user’s potential preferences regarding weekly meal planning or shopping; however, the diets also had to be balanced independently over the entire 35-day period. Each of the language models was asked, in separate prompts, to generate diets with energy values of 2,000 kcal, 2,500 kcal and 3,000 kcal. Each model was therefore intended to generate 105 days with different energy values (a total of 315 days’ worth of menus were generated). In cases where an incorrect number of days was generated (e.g., 5 days instead of 5 weeks), if there was a lack of relevant information regarding the weight or composition of a dish, or in the event of simplifications made by the LLM (e.g., instead of providing a menu for a specific day, stating that leftover ingredients from the week had been blended together), the model was asked to provide further details and/or to present a realistic 35-day plan meeting specific requirements. Once the meal plans had been generated, all diets were further reviewed and assessed by dietitian. Where any gaps were identified, the models were asked to provide further details regarding the proportions and composition of the dishes, so that there would be no doubt as to the nutritional value of the generated diets. Further enquiries were only raised when it became absolutely necessary to determine the nutritional values of the meals and diets generated. No changes were made intended to improve the nutritional quality of the meal plans. Any doubts regarding the product were resolved by a dietitian, taking into account the Polish context and the conditions specified in the prompt. To avoid discrepancies arising between the models when generating diets, all additional instructions were worded in a similar manner, using phrases such as ‘clarify’, ‘provide’, ‘present’ and ‘correct’. The commands were therefore as similar as possible across the LLMs. The procedure was described in the previous article (28), while the full, detailed prompts have been published in an open data repository^1^.

In addition to energy content, further requirements were set for the meal plans. The diets were to be generated for a healthy individual with no dietary restrictions, in good health and of normal body mass, so as not to restrict the models in their choice of specific products and dishes. This also simplified compliance with basic nutritional recommendations and enabled the generation of a diet with a specific, pre-set energy value, without the need to manipulate that value. All diets were also to be generated for a person with average cooking skills. The diets were to comply with Polish dietary recommendations (13, 14) and standards (29). In addition, the diet had to be generated for an average person aged between 19 and 30. This age range is dictated by the popularity of language models as user-assistance tools and also corresponds to one of the age groups specified in the nutritional standards for the Polish population (29). This allowed certain inaccuracies regarding the nutritional value of a specific nutrient to be avoided. Furthermore, the diet was intended to meet the criteria that are the subject of a major debate in contemporary society – to be environmentally friendly and sustainable, as well as tailored to the average food expenditure per capita in Poland. To achieve this, a range of average expenditure on food and drink was provided, based on the latest annual food data supplied by the Central Statistical Office (30). Meal plans should therefore be designed to suit the average Polish resident, who might use LLM to generate a diet plan.

### Assessment of the compliance of meal plans with polish healthy eating guidelines

The diets were assessed for compliance with nutritional recommendations for the Polish population (13, 14). The assessment criteria were developed on the basis of guidelines (general guidelines) and a three-step approach to their implementation (weight or proportions of individual product groups on a daily or weekly basis). Consequently, points were awarded for the introduction or restriction of selected food product groups or specific foods. The full scoring system is set out in Table 1. Salt intake was estimated on the basis of sodium intake. Sodium intake is shown in the nutritional analysis of the menu (28). The amount of salt consumed was calculated using the following formula:


Salt consumed=sodium consumedx2.5

**Table 1:** A scale for assessing the compliance of menus with the Healthy Eating Guidelines for the Polish population.

No.	Recommendation	Scale	Points
1	Eating wholegrain products with every meal	Daily	1
2	Consumption of fruit and vegetables should be at least 400 g a day (more vegetables than fruit)	Daily	1
3	Limiting the consumption of red meat and processed red meat products to 500 g per week	Weekly	7
4	Limiting salt intake to 5 g a day	Daily	1
5	Replacing sweets with fruit, nuts or seeds (no sweets)	Daily	1
6	Using vegetable fats instead of animal fats (reducing the intake of animal fats)	Daily	1
7	Eating a variety of oily fish twice a week	Weekly	7
8	Consume 2 portions of milk a day (or an dairy products such as yoghurt, kefir, buttermilk or cottage cheese)	Daily	1
9	Consumption of legumes as a meat substitute (the inclusion of legumes in the diet)	Daily	1

Daily recommendations (e.g., consuming 400 g of fruit or vegetables a day) were awarded 1 point each day. Weekly recommendations (e.g., limiting red meat consumption) were awarded 7 points for the whole week, with 1 point awarded for each day of the week on which the recommendation was met. Recommendations regarding the regularity of meals, physical activity, cooking methods or highly processed foods were not included, as it was not possible to verify these criteria in the context of a meal plan. It was therefore possible to score between 0 and 9 points each day which, in practice, meant the average number of recommendations implemented per day. All the recommendations were treated as equal, as the National Institute of Public Health did not single out any particular recommendations as being more important than others.

### Statistical analysis

Statistical analysis was performed using IBM SPSS Statistics v. 31.0.1.0 (IBM Corp., Armonk, NY, United States). The Shapiro–Wilk test was used to assess the normality of the data distribution. Due to the deviation of the distribution of total scores from a normal distribution in both groups and subgroups, only non-parametric tests were used in the analysis. The Kruskal–Wallis test was used to compare differences in the number of points scored between meal plans with different energy values between individual models. To assess the differences in the number of points scored between menus of different energy values within each model, a two-way Friedman analysis of variance for dependent samples with pairwise comparison was used. Differences between models in the number of days on which individual recommendations were met were compared using Pearson’s *χ*^2^ test. Significance levels were adjusted using the Bonferroni correction. Statistical significance was set at *α* = 0.05.

## Results

The results showing the scores achieved by individual models, according to their energy values, are presented in Table 2. The median score achieved by ChatGPT was 6.0 (mean 5.72 ± 0.7), while the median score achieved by Gemini was 5.0 (mean 5.30 ± 1.3) and the median score achieved by Copilot was 7.0 (mean 6.30 ± 1.2). Copilot scored higher, and thus demonstrated greater compliance with the Healthy Eating Guidelines for the Polish population, than ChatGPT (*p* = 0.002, Kruskal–Wallis test) and Gemini (*p* < 0.001, Kruskal–Wallis test). No statistically significant differences were observed between the ChatGPT and Gemini models (*p* = 0.093). When broken down by specific energy values, Copilot scored significantly higher for meal plans with target energy values of 2,500 kcal and 3,000 kcal than the other models (*p* < 0.05, Kruskal–Wallis test) and, for the meal plan with a target energy value of 2,000 kcal, significantly more points than ChatGPT (*p* = 0.014, Kruskal–Wallis test). No statistically significant differences were observed between the Gemini and ChatGPT models for any of the energy values, nor between the Copilot and Gemini models for the intended value of 2,000 kcal (*p* > 0.05, Kruskal–Wallis test).

**Table 2:** Score obtained by LLM models for compliance with the Healthy Eating Recommendations.

Target energy value	Number of points scored by LLMs			*p*-value
	ChatGPT [Mean ± SD Median Min-Max]	Gemini [Mean ± SD Median Min-Max]	Copilot [Mean ± SD Median Min-Max]	
2000 kcal	6 ± 1 6^a^ 4–8	6 ± 1 6 4–8	7 ± 1 7^a^ 5–8	^a^*p* = 0.014
2,500 kcal	6 ± 1 6^a^ 5–7	5 ± 1 5^ab^ 2–6	6 ± 1 6^b^ 4–8	^a^*p* < 0.001 ^b^*p* < 0.001
3,000 kcal	6 ± 1 6^a^ 4–7	5 ± 1 5^b^ 3–8	6 ± 1 7^ab^ 4–8	^a^*p* = 0.009 ^b^*p* = 0.002
OVERALL	5.72 ± 0.7 6.0^a^ 4–8	5.30 ± 1.3 5.0^b^ 2–8	6.30 ± 1.2 7.0^ab^ 4–8	^a^*p* = 0.002 ^b^*p* < 0.001

a, b – values marked with the same letters within rows differ significantly from one another – Kruskal–Wallis test.

When analyzing differences in meal plans with varying energy values within each model, no statistically significant differences were observed in ChatGPT between meal plans with different target energy values (*p* = 0.052, Friedman test). In the case of the Gemini model, it was found that meal plans with a target value of 2,500 kcal scored significantly more points than those with a target value of 2,000 kcal (*p* = 0.001, Friedman test). However, no statistically significant differences were observed in the other pairwise comparisons (*p* > 0.05, Friedman test). For the Copilot model, statistically significant differences were found between meal plans with different energy values (*p* < 0.001, Friedman test). Pairwise comparison showed that meal plans with a target value of 2,500 kcal scored significantly more points than those with a target value of 2,000 kcal (*p* = 0.015, Friedman test). No significant differences were observed for the remaining pairs (*p* > 0.05, Friedman test).

The number of days on which each recommendation was followed is shown in Table 3. Only recommendation 3, concerning the reduction of red meat consumption, was followed on all days by all LLMs. In addition, the models generally performed well with regard to recommendations 2 (adequate intake of fruit and vegetables), 5 (limiting the consumption of sweets), 6 (replacing animal fats with vegetable fats) and 8 (adequate intake of milk and dairy products). On average, the models performed moderately well in meeting recommendations 4 (limiting salt intake) and 9 (adequate intake of legumes) but failed to meet recommendation 1 (consumption of wholegrain products at every meal). Furthermore, Copilot met recommendation 7 (adequate consumption of oily fish) in all meal plans, while the ChatGPT and Gemini models met this recommendation in 4 and 5 weeks, respectively, (over a 15-week period). Statistically significant differences were observed between the models in terms of the number of days on which recommendations were followed for recommendation 1 (*p* = 0.012, *χ*^2^ test), 2 (*p* < 0.001, *χ*^2^ test), 4 (*p* = 0.002, *χ*^2^ test), 5, 6, 7, 8 and 9 (*p* < 0.001, *χ*^2^ test).

**Table 3:** Number of days on which the recommendations were implemented by each LLM.

Recommendation	ChatGPT	Gemini	Copilot	*p*-value*
Recommendation 1	0	6	9	*p* = 0.012
Recommendation 2	105	89	105	*p* < 0.001
Recommendation 3	105	105	105	N/A
Recommendation 4	19	42	34	*p* = 0.002
Recommendation 5	105	85	95	*p* < 0.001
Recommendation 6	103	76	78	*p* < 0.001
Recommendation 7	28	35	105	*p* < 0.001
Recommendation 8	90	81	62	*p* < 0.001
Recommendation 9	46	37	69	*p* < 0.001

*Pearson *χ*^2^ test.

## Discussion

To the author’s knowledge, this is the first study of its kind to examine the compliance of diets generated by AI tools with national dietary guidelines. It is also the first study of its kind to compare this compliance across different LLMs. It is also the first study to make these comparisons within a specific practical context – within the context of a given country, as well as from the perspective of a user without specialist knowledge of nutrition or experience of using LLM tools. This study therefore has a real impact on the assessment of the potential consequences of following these recommendations for the average user. In an era of growing popularity of LLM tools, this is an extremely important public health issue.

The median number of recommendations carried out per day was highest for Copilot and was significantly higher than for the other models. ChatGPT followed more recommendations per day than Gemini, although no statistically significant differences were observed. This means that the diets generated by Copilot were more consistent with polish healthy eating guidelines. A median compliance rate of 7 out of 9 recommendations is a relatively good result. It is worth noting that Copilot met more recommendations per day than the other models, regardless of energy intake. In previous studies, adherence to dietary recommendations has been linked to better health outcomes (31, 32). Given that the general public’s adherence to dietary guidelines is relatively low (4, 33–35), the use of such tools could improve adherence to dietary guidelines. It should be noted, however, that this compliance is only one of the factors that influence the healthiness of a diet. Compliance with the recommendations does not mean that other factors will also be met. As previous research from this project has shown, LLMs struggle to generate meal plans, particularly in terms of quantities, including the estimation of energy value (28). Greater adherence to the guidelines does not necessarily mean, therefore, that following these models will improve the healthiness of one’s diet, but it may point to certain measures, such as including more fish or reducing the amount of red meat and red meat products in one’s weekly meal plan.

It is worth noting that the energy content of the diet did not appear to have a noticeable impact on the extent to which the recommendations were followed. In the case of ChatGPT, no statistically significant difference was found between the generated menus with different energy values. Although, in the case of Copilot and Gemini, a diet with a planned energy intake of 2,500 kcal differed significantly from a 2000 kcal diet in terms of adherence to recommendations, no differences were found between planned energy intakes of 2000 kcal and 3,000 kcal, or between 2,500 kcal and 3,000 kcal. Any significant differences observed for the 2,500 kcal diet were most likely accidental. Although it might seem that a higher energy value could be associated with a greater number of daily recommendations being met – given the greater scope for introducing further products due to a certain energy reserve – all the models performed comparably across every energy range. It should be emphasized that this is a positive feature, indicating that dietary recommendations are being met to a comparable extent. The median compliance rate for each generated energy value, using each model, was greater than half. Given the low level of compliance with dietary recommendations among the general public, the use of these models may therefore have a positive impact; as mentioned earlier, Copilot performs best in this regard. It should be noted, however, that the ChatGPT and Gemini models showed moderate compliance. Differences between models in the number of recommendations met – and therefore in the degree of compliance with the recommendations – may stem from differences in their instruction-following capabilities. Models may differ in their ability to follow instructions and to adhere to task requirements in tasks involving lengthy contexts. Previous studies have shown that LLMs differ in their ability to carry out complex instructions, and the degree of compliance may vary depending on specific requirements within the same task (36, 37). While the LLMs generated meal plans that were largely consistent with selected recommendations – such as limiting the consumption of red meat and processed red meat products, eliminating sweets, increasing the proportion of fruit and vegetables, and reducing animal fats in favor of plant-based fats – relatively few meal plans complied with the recommendations regarding wholegrains. In the case of these products, Polish dietary guidelines suggest including them in every meal. Very few days met this recommendation. This does not mean, however, that the menus were unhealthy – the generated menus often included dairy products, such as yoghurt or buttermilk, with added fruit, for mid-morning or afternoon snacks. Clearly, frequent consumption of wholegrain products is associated with numerous health benefits, such as the prevention of certain cancers, cardiovascular diseases and diabetes, as well as weight management (38–40); however, positive health effects are observed even at much lower intakes, even below 100 g per day, with the health benefits increasing with intake (41–44). In their meta-analysis, Aune et al. (45) found that the health benefits of consuming wholegrain products increase up to a dose of 210–225 g/day. The recommendation to include a wholegrain product in every meal may therefore be slightly excessive and exceed this intake, and the health benefits of eating fruit and/or yoghurt instead of yet another wholegrain product may, in this case, prove to be greater, especially given the carbohydrates found in fruit and vegetables, which may be included in the meal. As demonstrated in previous studies, LLMs generated meal plans containing appropriate amounts of carbohydrates (28). While the solution presented by LLM appears reasonable in terms of health, the instruction clearly stipulated that the generated meal plans should comply with the dietary recommendations for the Polish population; in the case of wholegrain products, this requirement was not met.

The models also failed to meet the dietary recommendations regarding salt intake. The average table salt content in the meal plans, calculated on the basis of sodium content, was 6.32 ± 2.2 g/day, with a median of 6.27 g/day. Although this amount exceeds the recommended limit of 5 g/day, it should be noted that none of the meal plans suggested adding extra salt to dishes. These values therefore derive from the products included in the meal plans and do not consider the addition of salt to ready-made dishes or culinary processes such as cooking pasta, groats or potatoes. There is consequently a risk that consumers would add salt to selected products and dishes themselves while preparing them, and that their daily salt intake would increase significantly. While it is true that the daily salt intake observed in the general population is higher (46), this intake could be comparable following the aforementioned addition of salt to the generated meal plans. High intakes of sodium and salt in daily diets have been linked to an increased mortality rate, as well as to a number of diseases, primarily cardiovascular conditions (47–50). Reducing the consumption of products containing significant amounts of sodium would therefore be more beneficial for the generated meal plans.

Copilot also performed significantly better when it came to recommendations regarding the consumption of fish and pulses, which contributed to this LLM’s overall better performance. Frequent consumption of fish has been unequivocally linked in studies to numerous health benefits, including reduced mortality, a lower risk of certain cancers, cardiovascular disease, inflammatory bowel disease, metabolic syndrome, neurodegenerative diseases and depression, as well as a favorable lipid profile and improved musculoskeletal health (51–54). Studies indicate that the optimal frequency of consumption is twice a week (55, 56), which is in line with the recommendations. More frequent consumption of pulses has also been linked to reduced mortality and a lower risk of certain diseases (57–59). Compliance with these recommendations is a clear advantage of the Copilot model, while the ChatGPT and Gemini models failed to meet these health-related recommendations.

This study has both strengths and limitations. One of its advantages is that it examines the compliance of diets generated by LLMs with national dietary guidelines. To date, there have been no studies in this area. Another advantage is that this study is set within the context of a specific country. The specific nature of local dietary guidelines is important and far more relevant to the local population. Compliance with guidelines issued for a specific population is therefore crucial from the perspective of a country’s public health. An undeniable advantage is the generation of a sample diet plan simulating an average user who lacks the necessary knowledge to ask the right questions or to verify the answers received. This allows for an assessment of the potential impact of following such diets, in terms of both benefits and risks. Finally, an advantage is the presentation and analysis of numerous meal plans. Previous studies have focused on diets lasting a few days at most, none of which addressed the issue of compliance with dietary recommendations.

The limitations of this study should also be highlighted. Firstly, this study does not aim to test the maximum performance of the LLM. More precise questions, the provision of appropriate materials, or learning from its own answers and queries could result in the generation of better, more accurate diets that are more closely aligned with national dietary guidelines. However, this is also a strength of the study, as average users are rarely able to ask the right questions and often do not have the relevant materials they could provide to the LLM as a basis for creating diets with greater compliance with dietary recommendations. Secondly, only three LLM models were used. The use of other models could therefore yield different results. Thirdly, this study focuses on compliance with recommendations rather than on the potential impact of following these recommendations. It is therefore not possible to draw conclusions about a definitive positive or negative impact of the public’s use of these diets. It is worth emphasizing that all recommendations were treated as equally important. In some cases (e.g., specific diseases), individual recommendations may be more important than others. However, the aim of this study was to focus on the healthy population, and these findings should be applied to such individuals. Although the study was conducted with the utmost care and featured a unique design, the aforementioned strengths and limitations should be considered when interpreting the results.

## Conclusion

In conclusion, the compliance of the diets generated by the three most popular LLMs with national dietary guidelines was average, with the Copilot model generating diets that were more compliant. However, the generated meal plans can provide the average user with information on interesting ways to follow selected recommendations, such as reducing the consumption of red meat and processed red meat products, cutting out sweets, increasing the proportion of fruit and vegetables, or reducing animal fats in favor of plant-based fats. Following these diets may, however, result in salt intake exceeding dietary recommendations, or in the consumption of wholegrains in quantities inconsistent with the recommendations. Furthermore, the generated meal plans generally contained insufficient amounts of oily marine fish and legumes, although the Copilot model tended to generate diets with the correct proportion of these food groups. Given the lack of full alignment with the fundamental principles of healthy eating, the generated diets should not currently be followed by users, although in some cases they may lead to improvements in certain dietary patterns. Unsupervised use of LLM-generated diets by non-expert users may produce menus with average alignment with national healthy eating recommendations and should not replace professional dietary counseling.

## Data Availability

The datasets presented in this study can be found in online repositories. The names of the repository/repositories and accession number(s) can be found at: open data repository RepOD. Data: https://doi.org/10.18150/9GVUEG.

## Footnotes

1
https://doi.org/10.18150/9GVUEG
